# Predictors of retention in community-based methadone maintenance treatment program in Pearl River Delta, China

**DOI:** 10.1186/1477-7517-10-3

**Published:** 2013-03-06

**Authors:** Fang Yang, Peng Lin, Yan Li, Qun He, Qisui Long, Xiaobing Fu, Yulan Luo

**Affiliations:** 1 Institute for AIDS Prevention and Control, Center for Disease Control and Prevention of Guangdong Province, 176 Xin’gang Road West, Guangzhou, 510300, China

**Keywords:** Methadone maintenance treatment, Retention, Cox’s proportional hazards model

## Abstract

**Background:**

The aims were to identify predictors of treatment retention in methadone maintenance treatment (MMT) clinics in Pearl River Delta, China.

**Methods:**

Retrospective longitudinal study. Participants: 6 MMT clinics in rural and urban area were selected. Statistical analysis: Stratified random sampling was employed, and the data were analyzed using Kaplan-Meier survival curves and life table method. Protective or risk factors were explored using Cox’s proportional hazards model. Independent variables were enrolled in univariate analysis and among which significant variables were analyzed by multivariate analysis.

**Results:**

A total of 2728 patients were enrolled. The median of the retention duration was 13.63 months, and the cumulative retention rates at 1,2,3 years were 53.0%, 35.0%, 20.0%, respectively. Multivariate Cox analysis showed: age, relationship with family, live on support from family or friends, income, considering treatment cost suitable, considering treatment open time suitable, addiction severity (daily expense for drug), communication with former drug taking peer, living in rural area, daily treatment dosage, sharing needles, re-admission and history of being arrested were predictors for MMT retention.

**Conclusions:**

MMT retention rate in Guangdong was low and treatment skills and quality should be improved. Meanwhile, participation of family and society should be encouraged.

## Background

Heroin addiction is chronic relapsed encephalopathy and so far there is no treatment to cure. International evidence-based practices have proved that Methadone maintenance treatments (MMTs) are effective to reduce heroin use and high-risk behaviors, as well as to prevent transmission of HIV and HCV [1-4]. Since the appearance of HIV infection, MMTs have been pushed forward, and they have been proved helpful in reducing both drug use and the transmission of infectious diseases, such as HIV or viral hepatitis [5].

In consideration of epidemic of HIV among drug users, China initiated MMT in Yunnan, Sicuan, Zhejiang, Guizhou and Guangxi provinces in 2004 with 8 MMT clinics established .With the success of the pilot MMT clinics, 680 MMT clinics were established by the end of 2009 nationwide, 241975 patients were recruited accumulatively, and the retention rate was 65%. Meanwhile, 56 MMT clinics were established by the end of 2009 in Guangdong,19442 patients were recruited accumulatively, and the retention rate was 57.4%.The retention rate in Guangdong was low and a lot of patients had dropped out.

According to the Chinese Implementation Protocol for Community-Based Methadone Maintenance Treatment for Opiate Addicts, the eligibility criteria to participate in MMT were: (i) clients who are addicted to opiate according to addiction protocol; (ii) at least 20 years of age; (iii) the number of allowable missing treatment days was 7 consecutive days; and (iv) capable of complete civil liability.

Drug users testing HIV-positive needed only to fulfil requirement (i). Furthermore, a detailed clinical guideline for methadone treatment was added to the protocol to support clinical practice and comprehensive interventions were highlighted in the new protocol which suggested clinics offering ancillary services. These included counseling, psychosocial support, testing for HIV, syphilis, hepatitis C and tuberculosis, referrals for antiretroviral treatment, peer education, health education, group activities, social support and skills training for employment. The treatment fee for MMT services was not specified, as in some areas where heroin is easily obtained at low cost, the fee is reduced or even waived [6].

One principal target of MMT was to keep patients under treatment, by which, patients can receive treatment concerning psychology, behavior and personality. The patients dropped out are more likely to relapse or engage in high-risk behaviors contracting HIV [7].

The aim of this paper was to employ generalized survival in the analysis of MMT retention, to explore the factors associated with retention in MMT among drug users in Pearl River Delta region, Guangdong province.

## Methods

### Study sites and participants

Retrospective longitudinal study was carried out. There were a total of 56 MMT clinics in Guangdong until September 30^th^ in 2010, among which 31 clinics were located in Pearl River Delta region, 17 clinics in urban area and 14 clinics in rural area. Stratified random sampling was employed to select the clinics for this study. In accordance with the urban and rural classification, 3 clinics in urban and rural areas were selected respectively. Among the 6 clinics Liuhua Hospital of Shenzhen Prefecture, Third People’s Hospital of Foshan Prefecture and Guangzhou Prefectural Psychosis Hospital were in urban area; and Conghua Chronic Hospital of Guangzhou, Taihe Hospital of Taishan County and Xiaogang Hospital of Xinhui County were in rural area. All the patients (2728 individuals) who were enrolled between January in 2006 and September in 2010 in the selected MMT clinics in Pearl River Delta of Guangdong were recruited. Patients who were enrolled in other MMT clinics and temporally referred to the 6 clinics were not included in the present study.

### Data collection

The data including socio-demographic, testing, treatment and drug abuse history of the subjects in the 6 MMT clinics were obtained from the National AIDS Information System. All the databases were linked by the unique treatment serial number. Study variables included socio-demographic, drug taking history, clinical treatment, testing and illegal behaviors, etc.

The definition of retention encompassed : (1) patients remaining in MMT along the period of study; (2) patients who were temporarily referred to other MMT clinic other than the selected 6 clinics;(3) the period of study referred to duration between January 1^st^, 2006 and September 30^th^, 2010.

Retention duration is calculated from first MMT entry and up to the date patients dropped out of treatment or the end of the follow-up period (September 30^th^, 2010).

### Statistical analyses

Pearson X^2^ test was employed to analyze classified variables. The number of months in MMT clinic from first admission until the patient’s quit for treatment or until the end of follow-up was taken for calculating retention duration in treatment using Kaplan-Meier method. Life table method was used to calculate the cumulative retention rate. Cox’s proportional hazards model was employed to define the factors associated with retention in MMT. 21 independent variables including socio-demographic, drug taking history, clinical treatment, testing, follow up and illegal behaviors were enrolled in univariate analyses, of which variables significantly associated with retention (*p-*value < 0.05) were selected into the Cox regression multivariate analyses. Hazard Ratio (HR) and 95% confidence interval were presented. All independent variables are transformed into categorical variables. All analyses were done by using the SPSS-17.0 package.

## Results

### Sample description

The patients’ characteristics were detailed in Table 1. A total of 2728 patients were recruited, among which the mean age at first MMT entry was 36.4 years (S.D.=12.4 years). The majority of patients were males (72.8% compared to 27.2% females), 420 (15.4%)were less than 30 years, 1329 (48.7%)were unmarried, 1456 (53.4%) subjects were from urban area, 2181 (79.9%)were under elementary school educated, 1495 (54.8%) were unemployed, only a few of patients were in good relationship with family (18.0%), and 1293 (47.4%) living with family or friends. 560 (20.5%) initiated drug use less than 20 years old and 1537 (56.3%) had been taking drugs over 10 years. 322 (48.5%) had experience of being arrested. 567 (20.8%) subjects had been in treatment over 2 years, 1145 (42.0%) participants’ daily methadone treatment dosage was over 50mg, and 1288 (47.2%) perceived satisfaction with MMT services. 1103 (40.4%) subjects in treatment was tested positive for urine morphine. The accumulative retention rate was only 22.3% (607 individuals) (See Table 1).

**Table 1:** **Characteristics of Patients’ in methadone maintenance treatment clinics and relations to treatment retention (*n*=2728)**

**Variable**		**n**	**Proportion (%)**
Gender**	male	2448	89.7
	female	280	10.3
Age(years) *	≤30	420	15.4
	>30	2308	84.6
Marital status	single	1329	48.7
	married	1399	51.3
Education years**	<6	2181	79.9
	7-12	481	17.6
	>12	66	2.4
Employment	no	1495	54.8
	yes	1233	45.2
Relationship with family**	bad	2238	82.0
	good	490	18.0
Living with family or friends**	no	1435	52.6
	yes	1293	47.4
Initial drug use age	≤20	560	20.5
	21~30	1886	69.1
	>30	282	10.3
Re-admission	no	1406	51.5
	yes	1322	48.5
Daily drug use times pre-admission	≤3	2218	81.3
	>3	510	18.7
Drug use years pre-admission*	≤5	367	13.5
	6~10	816	29.9
	>10	1537	56.3
Needle sharing experience*	no	2333	85.5
	yes	395	14.5
Urban residence**	no	1272	46.6
	yes	1456	53.4
Daily dosage(mg)**	≤30	688	25.2
	31~50	895	32.8
	>50	1145	42.0
History of being arrested**	no	1406	51.5
	yes	1322	48.5
Perceived Satisfaction with MMT treatment**	no	1440	52.8
	yes	1288	47.2
Considering treatment open time suitable**	no	1458	53.4
	yes	1270	46.6
Urine morphine test**	negative	1625	59.6
	positive	1103	40.4
Considering treatment cost suitable**	no	1449	53.1
	yes	1279	46.9
Drug use cost pre-MMT admission	≤300	2122	77.8
	>300	606	22.2
Duration of MMT treatment(years) **	≤1	1616	59.2
	1 <&≤2	545	20.0
	>2	567	20.8
MMT treatment outcome	retained	607	22.3
	Drop-out	2121	77.7

Note: (*) For statistically significant differences to MMT retention *p*<0.05; (**) For statistically significant differences to MMT retention *p*<0.01.

### Univariate Cox’s proportional hazards model analyses

Retention as dependant variable and socio-demographic, drug taking history, clinical treatment, testing, follow up and illegal behaviors etc. as independent variables were introduced into the Univariate Cox’s proportional hazards model. The analyses showed that age group, residence in urban or rural area, income, living with family or friends, relationship with family, duration of taking drug prior to MMT admission, sharing needles, attitude towards treatment fee, attitude towards treatment open time, addiction severity(daily expense for taking drug pre-admission), re-enrolled history, treatment dosage, history of being arrested, communication with former drug taking peer last month, satisfaction toward MMT service were associated with MMT retention significantly (*p*<0.05) (see Table 2).

**Table 2:** Cox’s proportional hazards model analyses on factors associated with MMT retention duration

**Variables**	**Assignment**	**Univariate analyses**		**Multivariate analyses**	
		**HR**	**HR 95.0% CI**	**HR**	**HR 95.0% CI**
Age group(year)( ≤30=0)					
>30	1	0.69**	0.62~0. 77	0.78**	0.69~0.88
Residence(urban=0)					
rural	1	1.25**	1.20~1.30	1.12*	1.01~1.25
income(others=0)					
family or friends	1	0.19**	0.17~0.21	0.40**	0.31~0.52
fixed income	2	0.21**	0.18~0.24	0.40**	0.30~0.54
temporary income	3	0.20**	0.18~0.22	0.43**	0.33~0.55
social welfare	4	0.17**	0.11~0.26	0.41**	0.23~0.72
Living with family or friends(yes=0)					
other	1	5.50**	4.84~6.23		
Relationship with family(bad=0)					
good	1	0.31**	0.29~0.34	0.68**	0.58~0.80
Drug use years pre-admission(years) (>10=0)					
≤5	1	1.14*	1.02~1.28		
6~10	2	1.13**	1.04~1.23		
Sharing needles(no=0)					
yes	1	1.25**	1.12~1.39	1.23*	1.08~1.40
Considering treatment cost suitable(no=0)					
yes	1	0.35**	0.32~0.38	0.71**	0.60~0.84
Considering treatment operation time suitable(no=0)					
yes	1	0.27**	0.25~0.30	0.73**	0.62~0.87
Daily expense for drug (RMB¥)prior to MMT(>300=0)					
≤300	1	1.21**	1.10~1.33	0. 80**	0.71~0.90
Re-enrolled(no=0)					
yes	1	1.49**	1.42~1.56	1.41**	1.34~1.49
History of being arrested(no=0)					
yes		5.43**	4.96~5.96	1.35**	1.08~1.69
Daily treatment dosage(mg)(>50=0)					
≤30	1	1.25**	1.14~1.38	1.44**	1.29~1.61
31~50	2	1. 27**	1.17~1.39	1.33**	1.21~1.48
Communication with former drug taking peers last month(yes=0)					
no	1	0.60**	0.57~0.63	0.90**	0.84~0.98
Satisfaction with MMT service(no=0)					
yes	1	0.23**	0.20~0.25		

Note:MMT outcome assignment: Retention =0,drop-out=1.

* Statistically significant (p<0.05).

** Statistically significant (p<0.01).

### Multivariate Cox’s proportional hazards model analyses

The variables which were significantly associated with retention (P<0.05) in univariate Cox’s analyses were selected into multivariate Cox’s proportional hazards model analyses to define the predictors of MMT retention. The multivariate analyses showed that admission age over 30 years old (HR=0.78, 95% CI=0.69~0.88), keeping good relationship with family (HR=0.68, 95% CI=0.58~0.80), living on support from family or friends(HR=0.4, 95% CI=0.3~0.5), living on permanent income(HR=0.40, 95% CI=0.30~0.54), living on casual income(HR=0.43, 95% CI =0.33~0.55), living on social welfare(HR=0.41, 95% CI=0.23~0.72), considering treatment cost suitable (HR=0.71, 95% CI=0.60~0.84), considering treatment open time suitable (HR=0.73, 95% CI=0.62~0.87), daily cost for taking drug pre-admission less than 300 RMB¥ (HR=0.80,95% CI=0.71~0.90) and no communication with former drug taking peer within last month (HR=0.90, 95% CI=0.84~0.98) were protective factors for retention; whereas, living in rural area (HR=1.12, 95% CI=1.01~1.25), daily treatment dose less than 30 mg (HR=1.44, 95% CI=1.29~1.61), daily treatment dose is between 31–50 mg(HR=1.34, 95% CI=1.21~1.48), sharing needles(HR=1.23, 95% CI=1.08~1.40), re-admission in MMT(HR=1.41, 95% CI=1.34~1.49)and history of being arrested(HR=1.35, 95% CI=1.08~1.69)were risking factors for MMT retention (P <0.05) (see Table 2).

### MMT retention duration and retention rate

**Figure 1:**
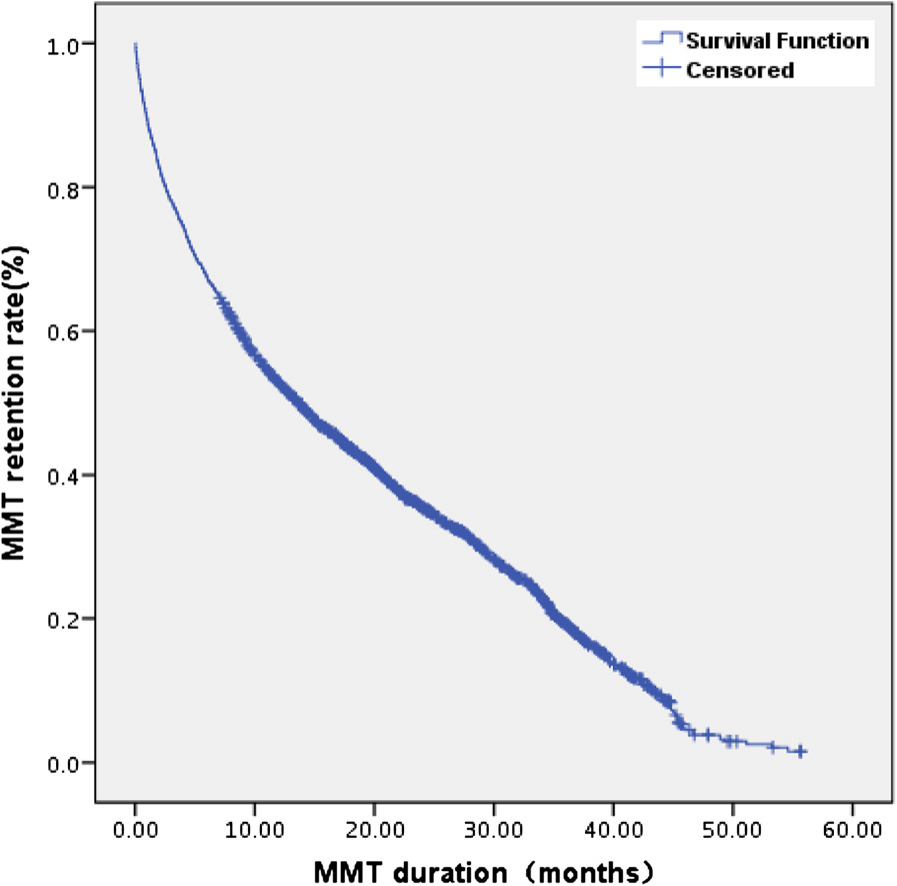
MMT retention rate and retention durance in Pearl River Delta, Guangdong, 2006.1-2010.9.

MMT retention duration was calculated by using Kaplan-Meier method. The median of the retention duration from the date when a patient first received MMT to the date when the patient dropped out or when the study expired(September 30th, 2010) was 13.63 months (see Figure 1). The minimum and maximum of the retention durations were 0.33 and 56.63 months, respectively. The cumulative retention rates were analyzed by life table method, and the rates at 1, 2, and 3 years were 53.0%, 35.0%, and 20.0%, respectively.

## Discussion

### Retention duration and retention rate

Retention rate and duration were the most important indicators to evaluate MMT [8]. This study showed that the median of retention duration was 13.63 months, which was less than the one (23 months) from the study in New York [9] but was longer than the ones from the studies in Italia (7 months) [10] and Urumqi (2.5 months) [7]. In addition, the retention duration in our study was consistent with the study in Spain and other sites [7,11,12]. While the cumulative retention rates at 1,2 and-3 years are 53.0%, 35.0% , 20.0%, respectively, which were far less than the ones shown by study carried out in Jiangsu province where retention rates at 1–2 years are 72.8%, 52.7% , respectively [13]. It was also less than 1 year retention rate (74.4%) shown by study in other country [14]. However it was longer than the study in Urumqi ( 46.9% after 9 months) [7,12]. There were a variety of factors which had effects on MMT retention rate [15]. The longer patients stayed in MMT clinic and no longer dependent on opioids, and receiving more supportive services, the more likely for patients to get better treatment outcome [9,16].

### Sociodemographic predictors for retention of MMT

Our study disclosed sociodemographic predictors for retention MMT included: age at admission over 30 years old, keeping good relationship with family, living on support from family or friends, living on permanent income, living on casual income, living on social welfare, daily cost for taking drug pre-admission less than 300 RMB¥, living in rural area, sharing needles and history of being arrested. All the predictors above were statistically significantly (*P*<0.05).

As age [6,17,18], income [17], addiction [19] and crime [9] were concerned, our findings were consistent with the studies carried out in other countries [6,9,17-19]. The common findings showed that age at admission in MMT over 30 years old, stable income, less crimes, no needle sharing and light addiction (daily expense for drug ≤300 RMB¥ pre-admission) were protective factors for MMT retention.

We found that criminal history was negatively associated with longer retention duration, and the group with history of being arrested had less retention duration than ones who had not. This finding was consistent with the study implemented in New York City (9).

Our study disclosed that the subjects lived in rural areas were more likely to drop out than those in urban areas. This was possibly due to lack of social supports and poor economic status in the countryside. There was a big difference between urban and rural areas not only in terms of sociodemographic factors but also the skills of medical staff in MMT clinics, which in turn influenced MMT maintenance retention.

Risking behaviors were negatively associated with MMT retention, In our study, sharing needles was found as risk factor for retention. The relationship should be further studied about the interactions between sharing syringe and the patients’ MMT related knowledge and attitude.

In terms of family ties and social support, correlated factors included keeping good relationship with family (HR=0.7, 95% CI=0.6~0.8), living on support from family or friends(HR=0.4, 95% CI=0.3~0.5), living on social welfare(HR=0.4, 95% CI= 0.2~0.7). This revealed the importance of family ties and social support as to MMT retention for patients. Family support could benefit the MMT clients in many different ways. Family members could help previous drug users to make important decisions, such as enrolling and remaining in the MMT programs. Thus, family support could also have significant implications for the efficacy of family-focused interventions and programming [20]. Potentially, family members could act as advocates for the policy, and encourage opioid users to participate in the treatment programs and take medications on a long-term, daily basis [20]. This echoed the findings in other research that, in order to be successful, HIV-related services and programs needed to involve families appropriately and effectively [21].

### Predictors for retention of MMT concerning MMT service

Predictors for retention of MMT concerning MMT service included: daily treatment dose less than 50 mg, re-admission in MMT were risk factors for MMT retention; while considering treatment cost suitable, considering treatment open time suitable, and no communication with former drug taking peer within last month were protective factors for MMT retention. All the predictors above were statistically significantly (Table 2).

Daily dosage attracted much more attentions and many studies were carried out to define the role of dosage towards retention rate. Our finding on the applicability of a high methadone dose to predict long-term retention replicated the experience of others [10,14,22]. The relationship among opinions about MMT clinic open time and treatment cost were also in accord with other studies initiated in other place [23,24].

On the contrary, our findings differed from those conclusions drawn from studies in other countries in terms of relationships between retention and experience of drop-out [23] and communication with former drug taking peers [7]. In our study, we found that having experience of drop-out and communication with former drug taking peers were risk factors for MMT retention.

Findings of this study should be considered in light of the following limitations. The data were drawn from a Pearl River Delta sample only. The MMT programs and clients in these areas might be different from those in other parts of Guangdong province. One should be cautious in generalizing the findings to other geographic locations and populations. Still, this study identified family and social supports, daily treatment dose, etc. as predictors of successful maintenance for methadone treatment.

## Conclusions

In conclusion, we found that most of our findings were in agreement with those of other studies in other countries and regions. Revealed by our study, what should be emphasized was that MMT retention was associated with not only treatment services, but also social supports. These findings highlighted that for sake of improvement of MMT retention and quality, we should not only improve skills of MMT medical staff but also improve family ties and social supports. Thus, for the service providers ongoing training on psychological counseling, behavior intervention, methadone pharmaceutics and dosage adjustment, etc. were in urgent need. On the other hand, family members and local communities should be recruited and educated to facilitate the process of MMT in order to achieve a higher rate of participation and compliance from community opioid addicts.

## Abbreviations

MMT: Methadone maintenance treatment

## Competing interests

The authors declare that they have no competing interests.

## Authors’ contribution

All authors were involved in the study concept and design. FY carried out data collection. FY, PL and YL performed statistical analyses and interpretation of the data. All authors participated in writing of manuscript. All authors read and approved the final manuscript.
